# Hemocompatibility of Emergency Bypass System versus Permanent Life Support extracorporeal membrane oxygenation in a propensity score-matched cohort: analysis of hematologic trajectories and transfusion requirements

**DOI:** 10.12701/jyms.2026.43.31

**Published:** 2026-05-07

**Authors:** Woo Sung Jang, Jung Uk Woo, Kyungsub Song

**Affiliations:** 1 Department of Thoracic and Cardiovascular Surgery, Keimyung University Dongsan Medical Center, Keimyung University College of Medicine, Daegu, Korea; 2 Department of Medicine, Keimyung University School of Medicine, Daegu, Korea

**Keywords:** Blood transfusion, Extracorporeal membrane oxygenation, Hemolysis, Thrombocytopenia

## Abstract

**Background:**

The Emergency Bypass System (EBS, Terumo Corporation) and Permanent Life Support (PLS, MAQUET Cardiopulmonary GmbH) platforms differ in pump design and surface coating, which may influence hemocompatibility. This study compared the longitudinal hematological profiles and transfusion requirements of these systems.

**Methods:**

Adult patients who underwent extracorporeal membrane oxygenation were analyzed using 1:1 propensity score matching (74 matched pairs). The primary endpoints included hemoglobin and platelet trajectories during the first 5 days, which were analyzed using linear mixed-effects models.

**Results:**

In the matched cohort, both groups demonstrated statistically similar longitudinal declines in hemoglobin and platelet counts (*p*=0.525 and *p*=0.501, respectively). However, this apparent stability in the EBS group was achieved at a significantly higher hematological cost. Within 5 days, the EBS group required 25.5% more red blood cell transfusions (*p*<0.001) and 34.2% more platelet transfusions (*p*<0.001) than the PLS group. Multivariable analysis confirmed that the use of the PLS system was independently associated with a lower transfusion demand.

**Conclusion:**

Although laboratory trajectories were comparable during the acute phase, patients supported by EBS experienced a substantially higher transfusion burden. These findings suggest a masking effect, in which compensatory transfusions may obscure the accelerated blood cell consumption associated with EBS. Therefore, clinicians should remain vigilant regarding the potential hidden hematologic costs of EBS support, even when laboratory parameters appear stable.

## Introduction

Extracorporeal membrane oxygenation (ECMO) is increasingly being used in patients with refractory cardiac and respiratory failure [1-3]. However, hematological complications, particularly thrombocytopenia and bleeding, are common and clinically significant. Previous research has suggested that blood exposure to artificial, nonbiological surfaces triggers systemic inflammatory responses and activates the coagulation cascade, resulting in consumptive coagulopathy [4]. In addition, the high shear stress generated by centrifugal pumps and turbulent circuit flow may cause mechanical hemolysis and proteolysis of von Willebrand factor, thereby increasing bleeding risk [5,6]. Adjunctive extracorporeal therapies may further influence platelet kinetics and bleeding risk; for example, concomitant continuous renal replacement therapy (CRRT) during ECMO has been associated with severe thrombocytopenia, defined as a platelet count <50,000/μL [7-11].

These hematologic disturbances are not merely transient laboratory abnormalities; major bleeding events and severe thrombocytopenia during ECMO support are strongly associated with increased mortality and poor neurologic outcomes [12, 13]. Although the technical aspects of ECMO delivery are increasingly recognized to influence clinical outcomes, evidence directly comparing the hemocompatibility of commonly used platforms is limited. The Permanent Life Support (PLS; MAQUET Cardiopulmonary GmbH, Rastatt, Germany) and Emergency Bypass System (EBS; Terumo Corporation, Tokyo, Japan) platforms differ in circuit components, surface coatings, priming volumes, and flow dynamics, which may differentially affect red blood cell (RBC) and platelet consumption, as well as overall transfusion requirements. However, comparative clinical data evaluating the longitudinal trajectories of anemia, thrombocytopenia, and bleeding outcomes between PLS and EBS remain scarce.

## Methods

**Ethics statement:** This study was conducted in accordance with the principles of the Declaration of Helsinki and was approved by the Institutional Review Board (IRB) of Keimyung University Dongsan Medical Center (IRB No: 2023-11-017-001). In the case of prospective participants, consent forms from all patients were collected. In the case of retrospective participants, the requirement for informed consent was waived due to the retrospective nature of the study. All methods were carried out in accordance with relevant guidelines and regulations.

### 1. Study design and outcomes

**Fig. 1.**
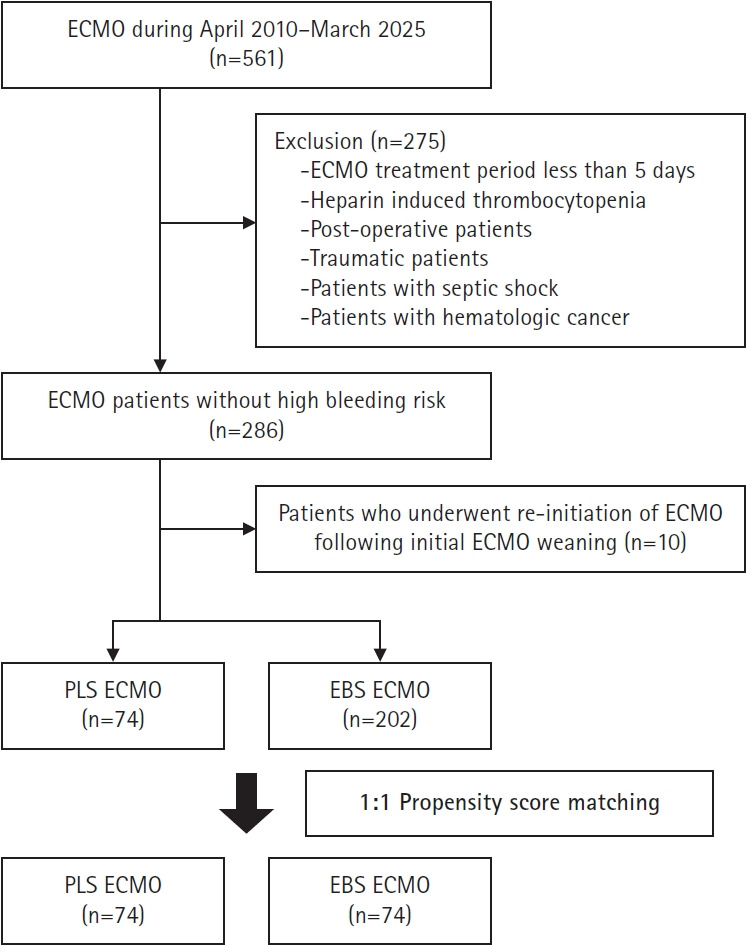
Flow diagram of patient selection. Patients were excluded if they meet any of the following criteria: ECMO treatment duration <5 days, heparin-induced thrombocytopenia, postoperative status, trauma, septic shock, hematologic malignancy, or reinitiation of ECMO after initial weaning. After applying these exclusions, the remaining patients were matched 1:1 between the two groups. ECMO, extracorporeal membrane oxygenation; PLS, Permanent Life Support (MAQUET Cardiopulmonary GmbH, Rastatt, Germany); EBS, Emergency Bypass System (Terumo Corporation, Tokyo, Japan).

This retrospective, single-center study included adult patients (aged >18 years) who received ECMO support. Patients were excluded if the ECMO duration was <5 days or if they had heparin-induced thrombocytopenia, postoperative status, traumatic injuries, septic shock, hematologic malignancies, or required recannulation. To minimize selection bias, a 1:1 propensity score matching (PSM) was performed between the PLS and EBS groups (Fig. 1).

Clinical data were evaluated during the first 5 days of support to capture acute hematologic responses while minimizing the confounding effects of long-term complications. The primary endpoint was the comparison of the longitudinal trajectories of hemoglobin and platelet counts between the groups. Secondary outcomes included bleeding complications and total RBC and platelet transfusion requirements.

### 2. Extracorporeal membrane oxygenation management and heparinization

Peripheral venoarterial (V-A) or venovenous ECMO was established using either the Seldinger technique or surgical cutdown, with the cannulation configurations determined according to clinical indications. Adjunctive procedures, including percutaneous atrial septostomy for left ventricular unloading and distal perfusion for limb ischemia, were performed when necessary. Systemic heparinization was administered to all patients without contraindications in accordance with the current guidelines. CRRT was integrated into the ECMO circuit; however, a separate dialysis catheter was inserted when circuit flow was insufficient to maintain adequate clearance.

### 3. Transfusion protocol

Transfusion practices followed the institutional protocol based on guidelines from the Extracorporeal Life Support Organization [10,14,15]. RBC transfusion was considered when hemoglobin levels fell below 7.0–8.0 g/dL or when signs of inadequate oxygen delivery were present. Platelet transfusion was initiated at counts of <50,000/μL in the absence of bleeding or <80,000/μL in the presence of active bleeding or planned invasive interventions.

### 4. Data collection and definitions

Hemoglobin levels and platelet counts were recorded daily during the first 5 days after ECMO initiation. Baseline hemoglobin levels and platelet counts were defined as the first laboratory values measured on day 0, immediately after ECMO initiation and stabilization. Severe thrombocytopenia was defined as a nadir platelet count of <50,000/μL within this time interval. Cumulative CRRT days were defined as the duration of concurrent renal replacement therapy during the 5-day study period. Cannulation-site bleeding was defined as a hemorrhage requiring intervention beyond manual compression, whereas bleeding-related mortality was defined as death primarily attributed to hemorrhage.

### 5. Statistical analysis

To minimize confounding factors, 1:1 PSM was performed using logistic regression based on baseline variables (Table 1), excluding dilated cardiomyopathy and prior cerebral infarction. Covariate balance was assessed using standardized mean differences (SMDs). Continuous and categorical variables were compared using the independent t-tests and chi-square or Fisher exact tests, respectively, with statistical significance defined as a two-tailed *p*-value <0.05.

Longitudinal changes in hemoglobin and platelet counts over the first 5 days were analyzed using linear mixed-effects models. In our model, we evaluated the fixed effects of the ECMO type, time, and the ECMO type×time interaction. The interaction term (ECMO type×time) was the key variable determining whether the rate of hematological decline differed between the PLS and EBS platforms. We also included the cumulative CRRT duration as a covariate to independently adjust for its impact on blood counts. Finally, we used random effects to mathematically account for individual differences in the starting baseline values and the paired nature of the matched patients. For missing daily laboratory values, the linear mixed-effects models were fitted using maximum likelihood estimation, which naturally accommodated unbalanced data without artificial imputation.

To analyze transfusion volumes, we utilized generalized linear models on log-transformed data (volume+1). To ensure the accuracy of this model, 32 patients who experienced overt bleeding complications were excluded from this specific analysis to prevent confounding by hemorrhagic events. Variables with a univariate *p*-value <0.20 were entered into the multivariable model to estimate independent associations, reported as adjusted percent changes. Additionally, to confirm the robustness of our primary findings and to evaluate whether the results remained consistent regardless of the ECMO modality, a subgroup analysis was conducted exclusively on patients receiving V-A ECMO. Within this specific subset, 1:1 PSM was independently performed using the aforementioned methodology, and all subsequent statistical procedures, including the linear mixed-effects and multivariable generalized linear models, were identically applied. All analyses were performed using R software (ver. 4.5.0; R Foundation for Statistical Computing, Vienna, Austria).

## Results

### 1. Matching

After applying the exclusion criteria, 276 patients were included: 202 in the PLS ECMO group and 74 in the EBS ECMO group (Fig. 1). After 1:1 PSM, 74 patients were included in each group. The distributions of propensity scores and SMDs for baseline covariates indicated an adequate balance between the groups (Supplementary Figs. 1, 2).

### 2. Patient characteristics

The mean ECMO duration in the overall cohort was 13.29±11.26 days. Specifically, the PLS group had a mean duration of 14.07±12.52 days, and the EBS group had a mean duration of 11.15±6.35 days (*p*=0.012). The mean age of the PLS group was 60.8±14.4 years, compared with 57.2±14.5 years in the EBS group (*p*=0.068). The PLS group had a significantly higher prevalence of chronic kidney disease (CKD) than the EBS group (26.2% vs. 13.5%, *p*=0.039) (Table 1).

After PSM (Table 2), baseline imbalances between the groups reduced substantially. ECMO duration was comparable (13.24±14.44 vs. 11.15±6.35 days, *p*=0.258), and there was no significant difference in the duration of CRRT during the study period (*p*=0.316) (Supplementary Table 1). Additionally, the prevalence of CKD (10.8% vs. 13.5%, *p*=0.801) and patient age (57.2±17.2 vs. 57.2±14.5 years, *p*>0.999) were well balanced. The SMDs for each variable are shown in Supplementary Fig. 2.

### 3. Comparison of longitudinal trends in hemoglobin and platelet counts during extracorporeal membrane oxygenation support

**Fig. 2.**
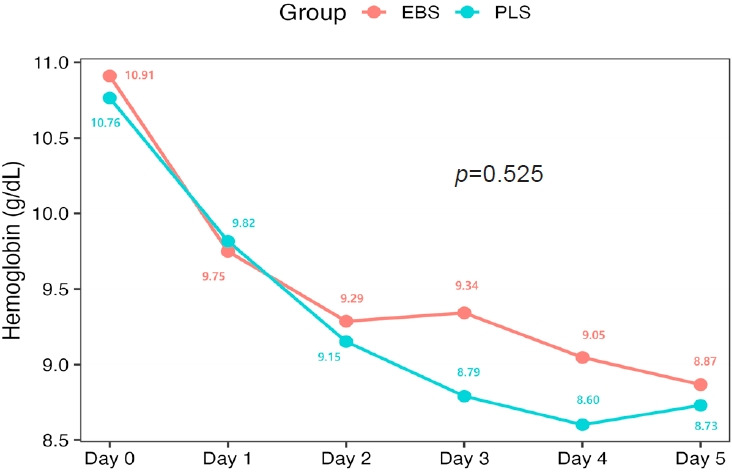
Comparison of serial changes in hemoglobin levels between the two groups. Linear mixed-effects models show that the rate of hemoglobin decline over time does not differ significantly between the PLS and EBS groups (*p*=0.525). PLS, Permanent Life Support (MAQUET Cardiopulmonary GmbH, Rastatt, Germany); EBS, Emergency Bypass System (Terumo Corporation, Tokyo, Japan).

In the matched cohort, longitudinal changes in hemoglobin levels were analyzed using linear mixed-effects models, accounting for repeated measurements within patients and matched pairs. Hemoglobin levels decreased significantly over time (β=−0.35 g/dL per day, *p*<0.001). The interaction between ECMO type and time was not statistically significant (*p*=0.525) (Fig. 2), indicating similar longitudinal hemoglobin trends in the PLS and EBS groups. CRRT duration was not independently associated with a decline in hemoglobin levels (*p*=0.865).

**Fig. 3.**
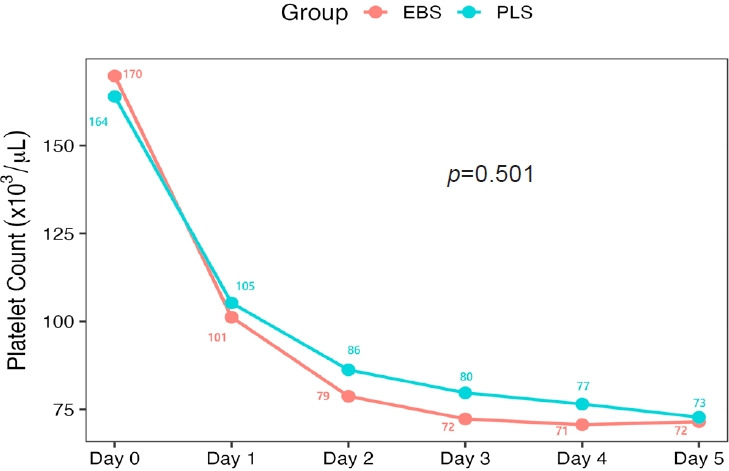
Comparison of serial changes in platelet levels between the two groups. Linear mixed-effects models show that the rate of platelet decline over time does not differ significantly between the PLS and EBS groups (*p*=0.501). PLS, Permanent Life Support (MAQUET Cardiopulmonary GmbH, Rastatt, Germany); EBS, Emergency Bypass System (Terumo Corporation, Tokyo, Japan).

Platelet counts also declined significantly over time in both groups (β=−17.2×10³/µL per day, *p*<0.001). No significant interaction between ECMO type and time was observed (*p*=0.501) (Fig. 3), suggesting similar platelet trends between PLS and EBS ECMO. In contrast, longer CRRT duration was independently associated with lower platelet counts (β=−4.7×10³/µL per day, *p*<0.001).

Furthermore, a subgroup analysis of patients without CRRT exposure (n=93) yielded consistent results; there were no significant interactions between ECMO type and time for the decline trajectories of either hemoglobin (*p*=0.864) or platelet counts (*p*=0.427).

### 4. Red blood cell and platelet transfusion comparison

During the first 5 days of ECMO support, the lowest and highest hemoglobin levels did not differ significantly between groups (lowest hemoglobin: 7.26±0.93 g/dL in the PLS group vs. 7.38±1.02 g/dL in the EBS group, *p*=0.481; highest hemoglobin: 12.92±2.26 g/dL in the PLS group vs. 12.65±1.87 g/dL in the EBS group, *p*=0.432) (Table 3). Similarly, the lowest and highest platelet counts were not significantly different (lowest platelet count: 53.54×10³±35.12×10³/μL vs. 48.14×10³±26.90×10³/μL, *p*=0.295; highest platelet count: 222.53×10³±81.16×10³/μL vs. 246.00×10³±117.38×10³/μL, *p*=0.159). However, patients in the EBS group required significantly more RBC and platelet transfusions than those in the PLS group (RBCs: 0.42±0.28 pack/ECMO day vs. 0.53±0.31 pack/ECMO day, *p*=0.022; platelets: 0.14±0.24 pack/ECMO day vs. 0.26±0.27 pack/ECMO day, *p*=0.005) (Table 3).

**Fig. 4.**
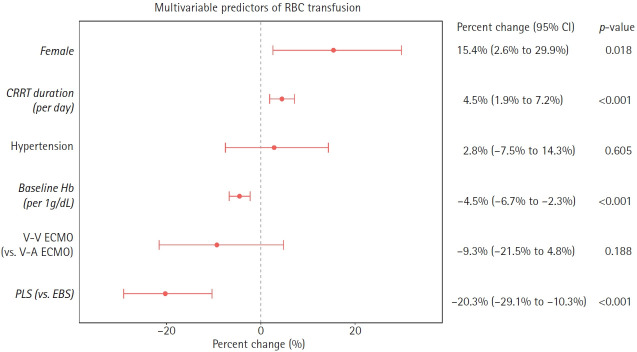
Multivariable generalized linear model results for red blood cell transfusion. RBC, red blood cell; CRRT, continuous renal replacement therapy; CI, confidence interval; Hb, hemoglobin; ECMO, extracorporeal membrane oxygenation; V-A, venoarterial; V-V, venovenous; PLS, Permanent Life Support (MAQUET Cardiopulmonary GmbH, Rastatt, Germany); EBS, Emergency Bypass System (Terumo Corporation, Tokyo, Japan).

**Fig. 5.**
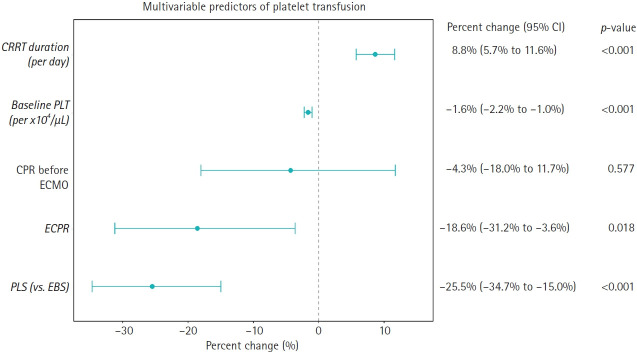
Multivariable generalized linear model results for platelet transfusion. CRRT, continuous renal replacement therapy; CI, confidence interval; PLT, platelet; CPR, cardiopulmonary resuscitation; ECMO, extracorporeal membrane oxygenation; ECPR, extracorporeal cardiopulmonary resuscitation; PLS, Permanent Life Support (MAQUET Cardiopulmonary GmbH, Rastatt, Germany); EBS, Emergency Bypass System (Terumo Corporation, Tokyo, Japan).

Multivariable generalized linear models demonstrated that use of the PLS ECMO system was an independent predictor of reduced transfusion requirements. Compared with the EBS, the PLS system was associated with a 20.3% reduction in RBC transfusions (95% confidence interval [CI], 10.3%–29.1%; *p*<0.001) and a 25.5% reduction in platelet transfusions (95% CI, 15.0%–34.7%; *p*<0.001) (Figs. 4, 5). Conversely, several factors were independently associated with increased transfusion volumes. Female sex was associated with a 15.4% increase in RBC requirements (95% CI, 2.6%–29.9%; *p*=0.018). Moreover, each additional day of CRRT support was associated with a 4.5% increase in RBC transfusions (95% CI, 1.9%–7.2%; *p*<0.001) and an 8.6% increase in platelet transfusions (95% CI, 5.7%–11.6%; *p*<0.001). Furthermore, higher baseline hematologic values were inversely associated with transfusion volume. A 1 g/dL increase in baseline hemoglobin level was associated with a 4.5% reduction in RBC transfusions (95% CI, 2.3%–6.7%; *p*<0.001). Similarly, a 10^4^/μL increase in baseline platelet count was associated with a 1.6% reduction in platelet transfusions (95% CI, 1.0%–2.2%; *p*<0.001). Finally, extracorporeal cardiopulmonary resuscitation cases were also associated with an 18.6% reduction in platelet transfusion volumes (95% CI, 3.6%–31.2%; *p*=0.018) (Figs. 4, 5).

### 5. Extracorporeal membrane oxygenation-related complications

The incidences of lower-limb ischemia (4.1% vs. 4.1%, *p*>0.999), sepsis (10.8% vs. 8.1%, *p*=0.779), cerebral infarction (5.4% vs. 6.8%, *p*>0.999), and ischemic colitis (1.4% vs. 1.4%, *p*>0.999) were comparable between the PLS and EBS ECMO groups. Overall bleeding complications did not differ significantly between the groups (5.4% vs. 13.5%, *p*=0.160). Individual bleeding events, including cannulation site bleeding (0% vs. 4.1%, *p*=0.245), gastrointestinal bleeding (1.4% vs. 4.1%, *p*=0.620), hemothorax (2.7% vs. 4.1%, *p*>0.999), and cerebral hemorrhage (1.4% vs. 4.1%, *p*=0.620), also showed no significant differences. Mortality due to bleeding complications (1.4% vs. 2.7%, *p*>0.999) was similar between the two groups (Table 4).

### 6. Subgroup analysis of patients who received venoarterial extracorporeal membrane oxygenation

To further validate these findings, a subgroup analysis was conducted exclusively on the patients supported by V-A ECMO. After 1:1 PSM within this subset, 65 matched pairs (n=130) were identified. Linear mixed-effects models demonstrated that there were no significant differences in the longitudinal decline trajectories of hemoglobin (*p*=0.848) and platelet counts (*p*=0.516) between the two systems. However, the PLS group required significantly lower transfusion volumes than the EBS group for both RBCs (0.42±0.27 vs. 0.54±0.32 packs/ECMO day, *p*=0.021) and platelets (0.13±0.22 vs. 0.27±0.27 packs/ECMO day, *p*=0.001).

In the multivariable generalized linear model for the V-A ECMO cohort, use of the PLS system remained an independent factor for reduced transfusion requirements. Compared to the EBS platform, the PLS system was associated with a 19.7% reduction in RBC transfusions (95% CI, 9.1%–29.1%; *p*=0.001) and a 28.8% reduction in platelet transfusions (95% CI, 17.9%–38.2%; *p*<0.001) after adjusting for baseline hematologic values and other clinical confounders. Overall, the subgroup findings were highly consistent with the results observed in the primary cohort.

## Discussion

The principal finding of this study was that while both ECMO systems demonstrated comparable longitudinal trajectories for hemoglobin and platelet counts, the EBS group incurred a substantially higher hematologic burden, requiring significantly greater volumes of RBC and platelet transfusions to maintain stability. This discrepancy suggests a potential association between EBS and a higher degree of blood component consumption, which is masked by compensatory transfusions. Consequently, these findings raise the possibility that EBS has a relatively inferior hemocompatibility profile. Importantly, this disparity emerged within the first 5 days of support during the initial phase of ECMO.

The observed differences in transfusion requirements were likely due to structural variations between the two ECMO systems. The PLS and EBS platforms differ in their pump designs, surface coatings, priming volumes, and flow dynamics [16-18]. Although both employ centrifugal pump technology, variations in impeller geometry and bearing mechanisms create distinct shear stress profiles. The PLS system (Rotaflow) features a sealless design with a magnetic levitation-like sapphire bearing, specifically engineered to minimize mechanical friction and heat generation during prolonged support [16,19]. By contrast, the EBS platform, which is optimized for rapid emergency deployment, may generate higher mechanical friction at the shaft seal or bearings. This effect is often clinically evident, as noticeable heat generation occurs within the pump drive unit. Previous studies have demonstrated a strong correlation between pump-generated heat and hemolysis, suggesting that thermal energy is a direct consequence of the mechanical shear forces that can induce sublethal erythrocyte damage and activate platelets [20–22]. In addition, differences in flow dynamics and surface coatings likely contributed to the discrepancy [17]. Collectively, these structural and thermal differences provide a plausible mechanistic basis for the association between the EBS platform and higher cellular consumption. Our multivariable analysis aligns with this interpretation, showing that the PLS system was independently associated with a 20.3% reduction in RBC transfusions and a 25.5% reduction in platelet transfusions compared with the EBS. This implies that without aggressive transfusion support, the EBS group would likely have experienced a significantly steeper decline in hematologic parameters, reflecting friction- and shear-induced injuries.

Despite the higher transfusion burden in the EBS group, clinical safety outcomes were comparable between the two systems. No significant differences were observed in major complications including bleeding events, ischemic events, survival to discharge, and weaning success rates. These findings support the clinical feasibility of EBS. However, the “hidden hematological costs” associated with EBS have important implications for resource management. Given the limited availability of blood products and the risks of transfusion, such as transfusion-associated circulatory overload and transfusion-related acute lung injury, clinicians should recognize the potential for increased blood product consumption when using the EBS platform. For patients with limited physiological reserves or those at a high risk of transfusion-related complications, ECMO system selection may represent an important clinical consideration.

Consistent with previous studies, our study reaffirmed the significant impact of concurrent CRRT on hematological parameters. Prolonged CRRT duration was independently associated with lower platelet counts and increased transfusion requirements for both RBCs and platelets. These findings align with those of previous studies by Buchtele et al. [9] and Tong et al. [23], who identified CRRT as a major contributor to thrombocytopenia in patients receiving ECMO. The incorporation of a hemofilter into an ECMO circuit increases the total artificial surface area and shear stress, thereby further accelerating platelet consumption, coagulation activation, and hemolysis.

This study has several limitations inherent to its retrospective single-center design. First, although PSM was employed to balance key prognostic factors and reduce selection bias, residual confounding factors may remain. Specifically, because of the emergent nature of ECMO cannulation, comprehensive pre-ECMO coagulation profiles (prothrombin time/international normalized ratio and activated partial thromboplastin time) were not consistently recorded. Furthermore, acute severity markers could not be incorporated into the PSM model because of the high incidence of missing data; for example, the vasoactive-inotropic score (VIS) and initial lactate levels were available for only 146 (52.9%) and 136 (49.3%) patients, respectively. Nevertheless, an analysis of this documented subset revealed no significant disparities in initial hemodynamic severity between the groups (mean VIS: 31.01±5.62 in the EBS group vs. 31.63±6.17 in the PLS group, *p*=0.556; mean initial lactate: 6.26±4.55 mmol/L in the EBS group vs. 5.24±4.74 mmol/L in the PLS group, *p*=0.244). Although these findings suggest that a gross systematic disparity in baseline severity is unlikely, the inability to fully match these parameters remains a potential confounding source. Second, regarding device allocation, ECMO device selection was determined by the immediate availability of equipment within the institution at the time of cannulation rather than randomization. Although this retrospective design lacks the inherent balance of a randomized controlled trial, we rigorously employed PSM and comprehensive multivariable adjustments to balance the baseline patient characteristics and control for potential confounders. We believe that this statistical approach effectively mitigates systematic bias and ensures a fair and independent comparison between the two systems. Third, our analysis was limited to the first 5 days of ECMO support. While this period captures the acute phase of device performance, it does not account for the long-term hematologic complications that may occur with prolonged support. Fourth, this study lacked direct biochemical indicators of hemolysis, such as lactate dehydrogenase, haptoglobin, and plasma-free hemoglobin levels. Future studies incorporating measurements of these specific biochemical parameters will provide crucial insights to objectively evaluate the hemolytic profiles of different ECMO platforms and definitively explain the underlying mechanisms. Finally, although transfusion practices followed institutional protocols, individual clinical decisions regarding transfusion triggers may have varied. Future prospective studies with longer follow-up periods, a comprehensive collection of acute severity markers, and detailed assessments of coagulopathy are warranted to validate these findings.

In summary, although both ECMO systems exhibited comparable longitudinal trajectories of hemoglobin levels and platelet counts during the acute phase, patients supported by EBS experienced a substantially higher hematologic burden. The markedly increased transfusion requirements in the EBS group, observed within the first 5 days of support, suggest a masking effect, whereby compensatory transfusions potentially obscured accelerated blood cell consumption. These findings indicate that the EBS platform may impose greater mechanical stress on hematological components than the PLS system does, underscoring the need for vigilant monitoring of transfusion demands, even when laboratory parameters appear stable.

## Figures and Tables

**Table 1.** Patients’ characteristics in the total cohort

Characteristic	Overall	PLS ECMO	EBS ECMO	*p*-value
No. of patients	276	202	74	
ECMO mode, venoarterial	234 (84.8)	169 (83.7)	65 (78.8)	0.505
ECMO time (day)	13.29±11.26	14.07±12.52	11.15±6.35	0.012^a)^
Male sex	185 (67.0)	137 (67.8)	48 (64.9)	0.750
Age (yr)	59.84±14.49	60.81±14.41	57.20±14.46	0.068
CPR before ECMO insertion	125 (45.3)	86 (42.6)	39 (52.7)	0.174
ECPR (%)	76 (27.5)	50 (24.8)	26 (35.1)	0.119
Comorbidity				
Coronary artery disease	47 (17.0)	36 (17.8)	11 (14.9)	0.691
Dilated cardiomyopathy	49 (17.8)	38 (18.8)	11 (14.9)	0.560
CKD	63 (22.8)	53 (26.2)	10 (13.5)	0.039^a)^
CKD on dialysis	19 (6.9)	17 (8.4)	2 (2.7)	0.164
Liver cirrhosis	15 (5.4)	12 (5.9)	3 (4.1)	0.766
Diabetes mellitus	113 (40.9)	78 (38.6)	35 (47.3)	0.245
Cerebral infarction	13 (4.7)	10 (5.0)	3 (4.1)	>0.999
Left atrial venting	28 (10.1)	22 (10.9)	6 (8.1)	0.650
Distal perfusion	57 (20.7)	47 (23.3)	10 (13.5)	0.108

Values are presented as number only, number (%), or mean±standard deviation. PLS, Permanent Life Support (MAQUET Cardiopulmonary GmbH, Rastatt, Germany); ECMO, extracorporeal membrane oxygenation; EBS, Emergency Bypass System (Terumo Corporation, Tokyo, Japan); CPR, cardiopulmonary resuscitation; ECPR, extracorporeal cardiopulmonary resuscitation; CKD, chronic kidney disease.

a) *p*<0.05, statistically significant.

**Table 2.** Patients’ characteristics following propensity score matching

Characteristic	Overall	PLS ECMO	EBS ECMO	*p*-value
No. of patients	148	74	74	
ECMO mode, V–A	128 (86.5)	63 (85.1)	65 (87.8)	0.810
ECMO duration (day)	12.20±11.16	13.24±14.44	11.15±6.35	0.258
Male sex	95 (64.2)	47 (63.5)	48 (64.8)	>0.999
Age (yr)	57.20±15.81	57.20±17.16	57.20±14.46	>0.999
CPR before ECMO insertion	77 (52.0)	38 (51.4)	39 (52.7)	>0.999
ECPR	50 (33.8)	24 (32.4)	26 (35.1)	>0.862
Comorbidity				
Coronary artery disease	21 (14.2)	10 (13.5)	11 (14.9)	>0.999
Dilated cardiomyopathy	24 (16.2)	13 (17.6)	11 (14.9)	0.824
CKD	18 (12.2)	8 (10.8)	10 (13.5)	0.801
CKD on dialysis	4 (2.7)	2 (2.7)	2 (2.7)	>0.999
Liver cirrhosis	4 (2.7)	1 (1.4)	3 (4.1)	0.620
Diabetes mellitus	68 (45.9)	33 (44.6)	35 (47.3)	0.869
Cerebral infarction	6 (4.1)	3 (4.1)	3 (4.1)	>0.999
Left atrial venting	15 (10.1)	9 (12.2)	6 (8.1)	0.586
Distal perfusion	25 (16.9)	15 (20.3)	10 (13.5)	0.380

Values are presented as number only, number (%), or mean±standard deviation. PLS, Permanent Life Support (MAQUET Cardiopulmonary GmbH, Rastatt, Germany); ECMO, extracorporeal membrane oxygenation; EBS, Emergency Bypass System (Terumo Corporation, Tokyo, Japan); CPR, cardiopulmonary resuscitation; ECPR, extracorporeal cardiopulmonary resuscitation; CKD, chronic kidney disease.

**Table 3.** Hemoglobin levels, platelet counts, and transfusion volumes during the first 5 days of ECMO support

Variable	Overall (n=148)	PLS ECMO (n=74)	EBS ECMO (n=74)	*p*-value
Severe thrombocytopenia^a)^(%)	86 (58.1)	39 (52.7)	47 (63.5)	0.244
Lowest hemoglobin (g/dL)	7.32±0.98	7.26±0.93	7.38±1.02	0.481
Highest hemoglobin (g/dL)	12.79±2.07	12.92±2.26	12.65±1.87	0.432
Lowest platelet count (×10^3^μL)	50.84±31.29	53.54±35.12	48.14±26.90	0.295
Highest platelet count (×10^3^μL)	234.26±101.25	222.53±81.16	246.00±117.38	0.159
Red blood cell transfusion (pack/ECMO day)	0.48±0.30	0.42±0.28	0.53±0.31	0.022^b)^
Platelet transfusion (pack/ECMO day)	0.20±0.26	0.14±0.24	0.26±0.27	0.005^b)^

Values are presented as number (%) or mean±standard deviation. ECMO, extracorporeal membrane oxygenation; PLS, Permanent Life Support (MAQUET Cardiopulmonary GmbH, Rastatt, Germany); EBS, Emergency Bypass System (Terumo Corporation, Tokyo, Japan).

a) Severe thrombocytopenia is defined as a platelet count of <50,000/μL.

b) *p*<0.05, statistically significant.

**Table 4.** ECMO-related outcomes in the propensity score-matched cohort

Variable	Overall (n=148)	PLS ECMO (n=74)	EBS ECMO (n=74)	*p*-value
Complications				
Lower limb ischemia	6 (4.1)	3 (4.1)	3 (4.1)	>0.999
Sepsis	14 (9.5)	8 (10.8)	6 (8.1)	0.779
Cerebral infarction	9 (6.1)	4 (5.4)	5 (6.8)	>0.999
Ischemic colitis	2 (1.4)	1 (1.4)	1 (1.4)	>0.999
Bleeding complications	14 (9.5)	4 (5.4)	10 (13.5)	0.160
Cannulation-site bleeding	3 (2.0)	0	3 (4.1)	0.245
Gastrointestinal bleeding	4 (2.7)	1 (1.4)	3 (4.1)	0.620
Hemothorax	5 (3.4)	2 (2.7)	3 (4.1)	>0.999
Cerebral hemorrhage	4 (2.7)	1 (1.4)	3 (4.1)	0.620
Mortality due to bleeding	3 (2.0)	1 (1.4)	2 (2.7)	>0.999
Survival to discharge	68 (45.9)	38 (51.4)	30 (40.5)	0.248
ECMO weaning	83 (56.1)	45 (60.8)	38 (51.4)	0.320

Values are presented as number (%) or mean±standard deviation. ECMO, extracorporeal membrane oxygenation; PLS, Permanent Life Support (MAQUET Cardiopulmonary GmbH, Rastatt, Germany); EBS, Emergency Bypass System (Terumo Corporation, Tokyo, Japan).

## Supplementary materials

Supplementary Figs. 1, 2 and Supplementary Table 1 can be found at https://doi.org/10.12701/jyms.2026.43.31.

Supplementary Fig. 1. Propensity score distribution (A) before and (B) after matching between the Emergency Bypass System (EBS; Terumo Corporation, Tokyo, Japan) and Permanent Life Support (PLS; MAQUET Cardiopulmonary GmbH, Rastatt, Germany) groups. ECMO, extracorporeal membrane oxygenation.

Supplementary Fig. 2. Standardized mean differences in baseline variables before and after propensity score matching. CKD, chronic kidney disease; ECPR, extracorporeal cardiopulmonary resuscitation; ECMO, extracorporeal membrane oxygenation; CPR, cardiopulmonary resuscitation; DM, diabetes mellitus; CAD, coronary artery disease.

Supplementary Table 1. Duration of CRRT during the study period
