# Supplementary material for: Hemocompatibility of Emergency Bypass System versus Permanent Life Support extracorporeal membrane oxygenation in a propensity score-matched cohort: analysis of hematologic trajectories and transfusion requirements
Source: J Yeungnam Med Sci. 2026 May 7;43:31. doi: 10.12701/jyms.2026.43.31 (PMC13373687; doi:10.12701/jyms.2026.43.31)
Supplement: Supplementary Fig. 1. — Propensity score distribution (A) before and (B) after matching between the Emergency Bypass System (EBS; Terumo Corporation, Tokyo, Japan) and Permanent Life Support (PLS; MAQUET Cardiopulmonary GmbH, Rastatt, Germany) groups. ECMO, extracorporeal membrane oxygenation. [file jyms-2026-43-31-Supplementary-Fig-1.pdf]

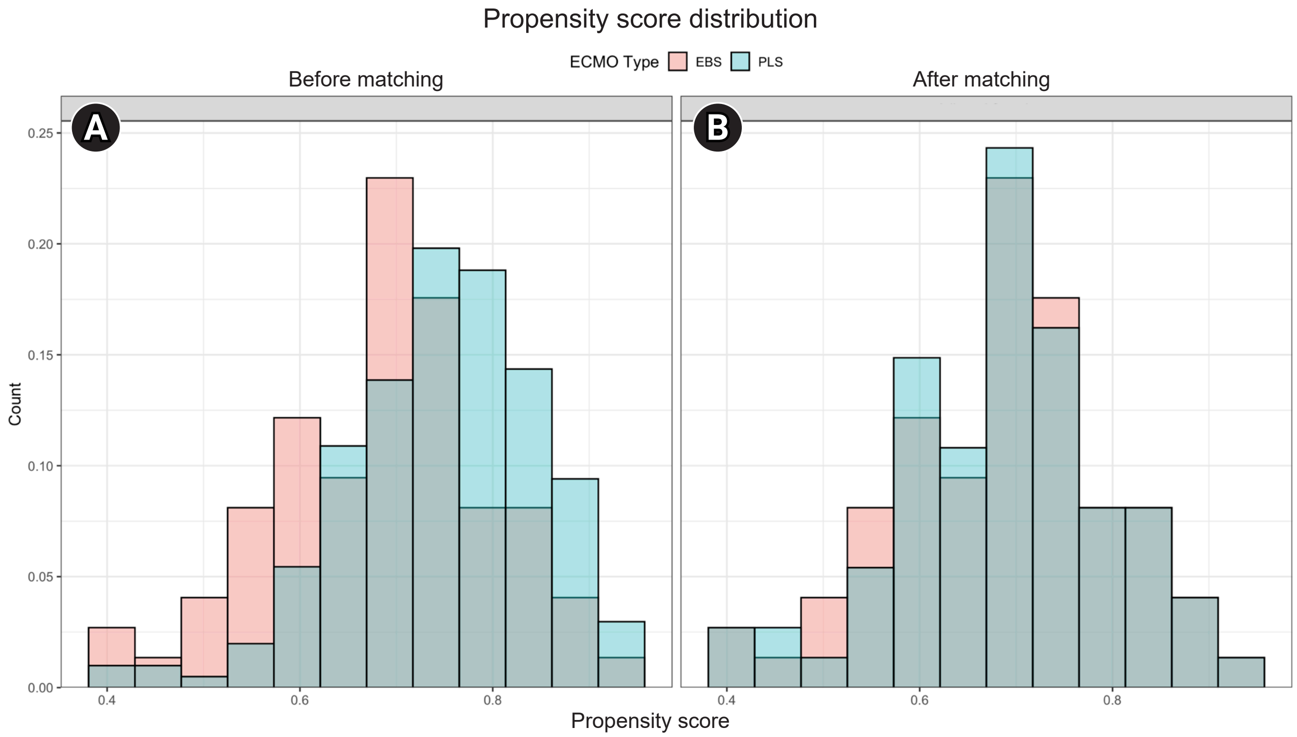

Supplementary Fig. 1. Propensity score distribution (A) before and (B) after matching between the Emergency Bypass System (EBS; Terumo Corporation, Tokyo, Japan) and Permanent Life Support (PLS; MAQUET Cardiopulmonary GmbH, Rastatt, Germany) groups. ECMO, extracorporeal membrane oxygenation.
