# Supplementary material for: Hemocompatibility of Emergency Bypass System versus Permanent Life Support extracorporeal membrane oxygenation in a propensity score-matched cohort: analysis of hematologic trajectories and transfusion requirements
Source: J Yeungnam Med Sci. 2026 May 7;43:31. doi: 10.12701/jyms.2026.43.31 (PMC13373687; doi:10.12701/jyms.2026.43.31)
Supplement: Supplementary Fig. 2. — Standardized mean differences in baseline variables before and after propensity score matching. CKD, chronic kidney disease; ECPR, extracorporeal cardiopulmonary resuscitation; ECMO, extracorporeal membrane oxygenation; CPR, cardiopulmonary resuscitation; DM, diabetes mellitus; CAD, coronary artery disease. [file jyms-2026-43-31-Supplementary-Fig-2.pdf]

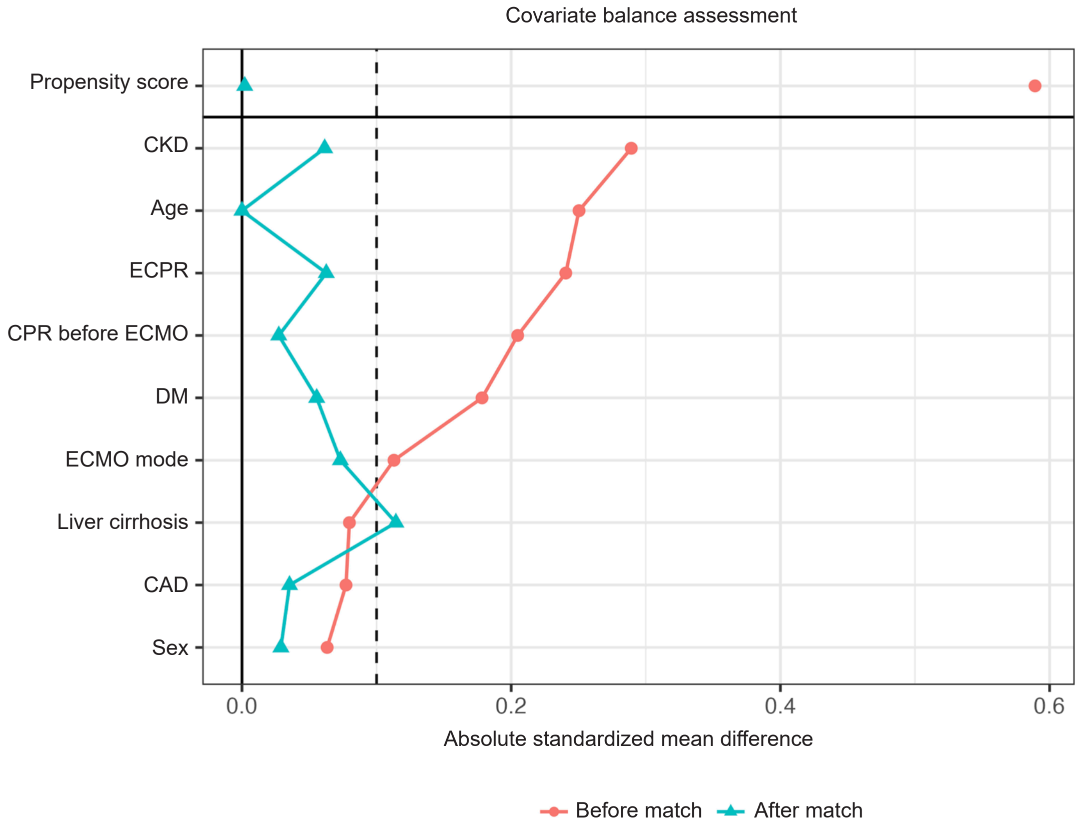

Supplementary Fig. 2. Standardized mean differences in baseline variables before and after propensity score matching. CKD, chronic kidney disease; ECPR, extracorporeal cardiopulmonary resuscitation; ECMO, extracorporeal membrane oxygenation; CPR, cardiopulmonary resuscitation; DM, diabetes mellitus; CAD, coronary artery disease.
