# Supplementary material for: Hemocompatibility of Emergency Bypass System versus Permanent Life Support extracorporeal membrane oxygenation in a propensity score-matched cohort: analysis of hematologic trajectories and transfusion requirements
Source: J Yeungnam Med Sci. 2026 May 7;43:31. doi: 10.12701/jyms.2026.43.31 (PMC13373687; doi:10.12701/jyms.2026.43.31)
Supplement: Supplementary Table 1. — Duration of CRRT during the study period [file jyms-2026-43-31-Supplementary-Table-1.pdf]

Supplementary Table 1. Duration of CRRT during the study period

CRRT duration	Overall (n = 148)	PLS ECMO (n = 74)	EBS ECMO (n = 74)
No CRRT	93 (62.8)	52 (70.3)	41 (54.1)
1 day	1 (0.7)	0	1 (1.4)
2 days	5 (3.4)	2 (2.7)	3 (4.1)
3 days	7 (4.7)	3 (4.1)	4 (5.4)
4 days	9 (6.1)	2 (2.7)	7 (9.5)
5 days	33 (22.3)	15 (20.3)	18 (24.3)

Values are presented as number (%) or mean ± standard deviation.
CRRT, continuous renal replacement therapy; PLS, Permanent Life Support System (MAQUET Cardiopulmonary GmbH, Rastatt, Germany); ECMO, extracorporeal membrane oxygenation; EBS, Emergency Bypass System (Terumo Corporation, Tokyo, Japan).
The duration of CRRT during the study period did not differ significantly between the groups ($p = 0.316$).
